# Dental extraction following zoledronate, induces osteonecrosis in rat´s jaw

**DOI:** 10.4317/medoral.21609

**Published:** 2017-02-04

**Authors:** Ximena Vidal-Gutiérrez, José-Francisco Gómez-Clavel, Luis-Alberto Gaitán-Cepeda

**Affiliations:** 1 Laboratory of Clinical and Experimental Pathology, Graduate and Research Division, Dental School, National Autonomous University of Mexico. Circuito Institutos s/n, Ciudad Universitaria, 04510 Coyoacán, D. F. Mexico city, México; 2 Laboratory of Dental and Education Research, Faculty of Superior Studies-Iztacala, National Autonomous University of Mexico. Av. De Los Barrios No. 1, Col. Los Reyes Iztacala, C.P. 54090, Tlalnepantla, Estado de México

## Abstract

**Background:**

Bisphosphonate-Related Osteonecrosis of the Jaw (BRONJ) is clinically characterized by the presence of exposed bone in the oral cavity that persists for more than eight weeks. Previous attempts to establish an animal model have not sufficiently considered disease features. Our aim was to establish an inexpensive and replicable animal model that develops BRONJ in a short time.

**Material and Methods:**

Thirty-two male Wistar rats were randomly divided into two groups: control and experimental. In the experimental group, we administered 0.06mg/kg intraperitoneal dose of zoledronic acid (ZA) 7 and 14 days prior to maxillary second molar extraction. At two, four and six weeks after tooth extraction, the animals were euthanized, and we dissected the maxilla following histological procedures. We stained serial slides with hematoxylin and eosin and Masson’s trichrome. The samples were harvested for macroscopic, radiologic and histological evaluation of bone changes.

**Results:**

At two weeks postextraction, we observed exposed necrotic bone in dental socket areas in experimental groups. Radiological analysis revealed osteolytic lesions accompanied by extensive destruction and sequestrum formation in the same group. Histological examination confirmed the absence of necrotic bone in control groups in contrast with the experimental groups. The percentage of empty lacunae and the number of osteoclasts and the necrotic bone area were significantly increased (*p*<0.05) in the experimental groups.

**Conclusions:**

The animal model using ZA administration to prior dental extraction successfully mimicked human BRONJ lesions. Also, the model was easily replicated, inexpensive and showed different features than other previous BRONJ models.

**Key words:**Bisphosphonates, osteonecrosis, dental extractions, animal model, BRONJ.

## Introduction

Bisphosphonates (BPs) represent a major class of antiresorptive drugs used in the prevention and treatment of osteoporosis, hypercalcemia of malignancy, multiple myeloma, Paget’s disease and bone metastases associated with breast, prostate, and lung, as well as osteogenesis imperfecta, fibrous dysplasia, and Gaucher’s disease (1,2). In afflicted patients, it effectively returns bone mineral density, reduces the incidence of bone fracture, and improves their quality of life. Despite these beneficial effects, a growing number of patients on long-term and high-dose BP therapy develop a potentially serious complication: osteonecrosis of the jaw (3,4).

BRONJ is defined clinically as an area of exposed bone in the maxillofacial region that does not heal within eight weeks in patients with no history of radiation therapy to the head and neck region. In this condition, the bone tissue in jaws often fails to heal following minor trauma, although it can also occur spontaneously (5-8). The most commonly reported symptoms of this condition are bone exposure, pain, bone sequestration, ﬁstulas that extend from mucous membranes to the surface of the skin, edema, paraesthesia and susceptibility to pathological bone fractures (9). However, in 90% of cases, it is caused by dental treatment of some kind.

Most cases of BRONJ have been reported in patients receiving prolonged intravenous treatment of zoledronate. BRONJ is rare in patients taking oral BPs for osteoporosis, and it is estimated between 0.001% and 0.1%. A recent study revealed an increased number of BRONJ cases, possibly owing to chronic oral BP use and thorough screening (1,4,10).

Several research groups and medical societies have recently published recommendations or guidelines on prevention, staging, and management strategies for BRONJ (11). Despite this, there is still a lack of information concerning the incidence, pathogenesis, treatment strategies, and prevention of BRONJ.

Numerous BRONJ studies have been previously performed on rats, but their results were inconsistent. For example, these researches have different protocols from the dose, interval, duration, route of administration of the BP medication to the use of co-medications for BRONJ induction had been used (12-17). Furthermore, in previous studies, their findings are not reliable and comparable, so the development of an inexpensive easily replicable experimental animal model is necessary to help to understand the physiopathology of BRONJ and eventually to establish preventive strategies and better treatment of this lesion.

Our goal was to develop a clinically relevant, inexpensive, and easily replicable animal model of Wistar rats treated with zoledronic acid and subjected to oral surgery in the form of dental extraction.

## Material and Methods

- Animals

We used thirty-two male Wistar rats (Rattus norvegicus, albinus) weighing from 200-250g which were obtained from the bioterium of the National Autonomous University’s Faculty of Higher Studies in Iztacala, Mexico. The rats were housed in cages at an ambient temperature (21°C-27°C) with a 12h light/dark cycle. Animals were fed a standard diet of rat chow and drink tap water ad libitum. The research protocol was conducted following the strict federal guidelines for the use and care of laboratory animals (NOM-062-ZOO-1999). The research protocol was submitted and approved by the Ethical Committee from Faculty of Superior Studies- Iztacala, National Autonomous University of Mexico, Mexico (PE209312).

- Study Design

We randomly divided the rats using a random number table into two groups (n=16 per group): the control and the experimental group. We divided the both study groups in three subgroups regarding the sacrificed moment: two weeks (n=6), four weeks (n=6) and six weeks (n=4) after dental extraction. An allocation concealment was performed.

- Study Procedures

- Dosage

We established the dose of zoledronic acid (Zometa®, Novartis Pharma©, Basel, Switzerland) at 0.06mg/kg, according to previous studies (18) and the experimental group was administered intramuscularly to the seventh and fourteenth day before dental extraction. At the same time, the control rats received the same volume of saline solution.

- Dental extractions and post-surgical care

We extracted the second maxillary molar, as follows: We administrated intramuscularly animals previously anesthetized with ketamine (60mg/kg) and xylazine (7mg/kg), once verified sedation, we performed the oral disinfection and then we detached the gingiva around the second maxillary molar using a sharpened dental instrument. Later the luxation and extraction of the second maxillary tooth were done using a modified veterinary dental instrument for rodent animals (iM3® Cat. No D2001).

We continuously monitored the cardiac frequency and body temperature during all surgical procedures, following the ethical standards of care for experimental animals. Surgical events as surgery’s length, dental fracture, residual roots and alveolar lamina fracture, were recorded.

All of pre-surgical, surgical and post-surgical cares were done according to NOM-062-ZOO-1999 federal guidelines for the use and care of laboratory animals.

**Figure 1:**
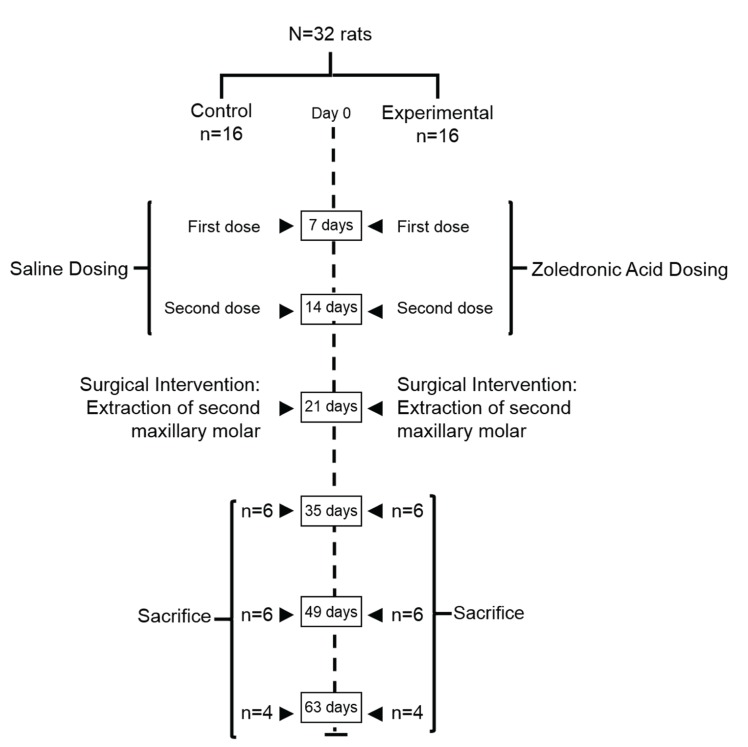
Experimental design for the study.

Animals from each study group were euthanized by over exposure to CO2 at two, four and six weeks posterior to dental extraction (Fig. 1).

Immediately after their sacrifice, samples of all animals was dissected, photographed, and radiographed using a standard dental X-ray. All samples were fixed in 10% buffer formaldehyde by 72 h minimum. Later all samples to posteriorly decalcify in 2% HNO3 for 48h. After decalcification period, all samples were embedded in paraffin to be cut at 5μm thick. Samples were sagittally sectioned. Three histological slides per sample were obtained and stained with hematoxylin and eosin technique to be used for histological analysis. In three random samples per group, additionally, three central sections were stained with Masson’s trichrome for cross-reference. We used a light microscopy (Leica DM750) for histological examination, and photomicrographs were taken using the Leica LAS-EZ software.

- Macroscopic Analysis

We obtained the follow data from the macroscopic evaluation of dissected maxilla: open socket, exposed bone, loss of adjacent teeth, and bone fracture. We used these data to determine the clinical presence of BRONJ as long as present at least three of the four conditions.

- Radiological Analysis

The X-ray images were obtained from the maxilla dissected immediately sacrifice to all animals. The standardized radiological parameters for all study groups were 60-70kV with an exposure time of 0.24 seconds. X-ray images were analyzed according to Pacheco using the IMAGE J software® (9). All images were converted into an 8-bit data (256 grey levels) generating a scale (0-255) for each image. The value zero represented the lowest and 255, the highest attenuation of X-ray beams. Image density analyses were conducted using grey levels of the animals alveolar bone tissue.

- Histological Analysis

**Figure 2:**
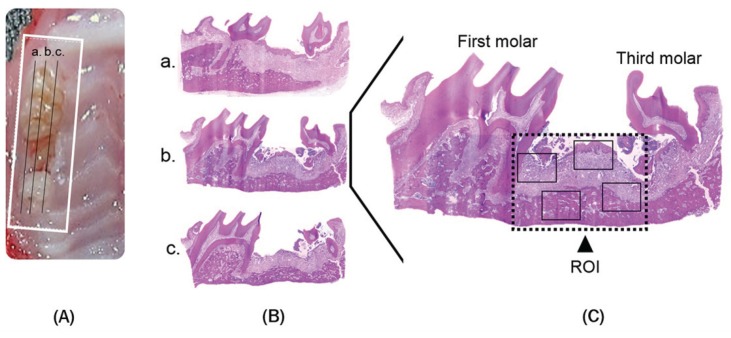
Histological Analysis. The samples of maxilla were taken by white square (A) and sagittally sectioned (B) in three areas of interest: vestibular (a.), middle (b.) and Palatine (c.); two samples from each area were evaluated in Hematoxylin and Eosin stain. Histological evaluation was performed in 4 zones (black squares) of the region of interest (ROI) randomly chosen (C).

We considered that the distance between distal first molar root to mesial third molar root was the region of interest (ROI), and then we assessed on the hematoxylin-eosin & Masson’s trichrome stained sections. For each animal/socket, histological slide sections from the lingual, medial and vestibular area were evaluated; we considered four zones of ROI by histological features, and they were photographed at 40x using a binocular light microscope Leica DM3000 (Fig. 2). We superposed a grid image with 36 points on histological fields per histological section. The grid image comprised of all tooth sockets from the coronal limits adjacent to the gingival epithelium until the lower apical limit.

Histological changes were evaluated using the following qualitative criteria: the presence of ulcerative lesion with exposed and necrotic bone, osteolysis, presence of hyperplasia of the epithelium accompanied by inflammatory cell infiltration, and the presence of sequestrum and bacterial colonies. (6,7,12,13).

Also, we obtained quantitative data, included gingival tissue repair area (Tissue, mm2), empty lacunae (#/mm2), presence and quantity of osteoclasts (#/mm2). We defined osteonecrosis as a loss of more than five contiguous osteocytes with confluent areas of empty lacunae. The analysis was performed in 4 zones of ROI, stained with Masson’s trichrome to visualize fibrous network in bone marrow areas and evaluate the organization of bone matrix.

All measurements were performed by a blinded observer previously calibrated.

- Statistical Evaluation

Data were expressed as mean ± standard derivation (SD). We determinate the normal distribution and homogeneity for data before statistical analyses. Data were then analyzed using ANOVA and Student’s t-test. For data that did not fit in the distribution of normality, we used the Mann-Whitney and Kruskal-Wallis tests. Values of *p*<0.05 were considered statistically significant. To establish possible associations between each group we used bivariate Xi2 tests with a 95% signiﬁcance level (*p*<0.05). We used STATA 12 for Windows (StataCorp LP., Lakeway, Texas, USA) to analyze the data.

## Results

**Figure 3:**
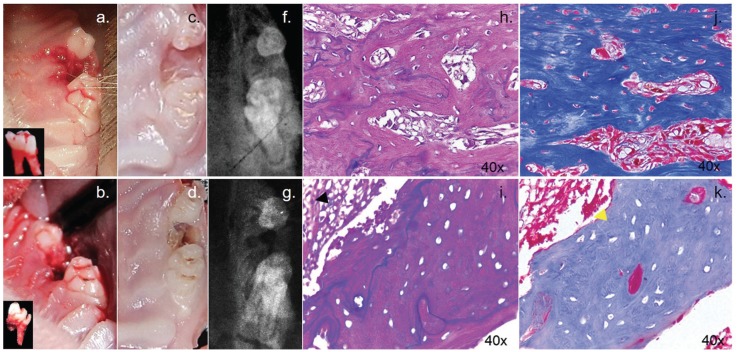
Observation of specimens at different times. Immediately after dental extraction, the control group shows a complete extracted molar (a.); in contrast, the experimental group shows a fractured root (b.). The rats sacrificed at six weeks after extraction in control group shows complete healing of oral mucosa (c.) the experimental group shows incomplete healing of oral mucosa, showing exposed alveolar bone (d.). Radiographically the control group (f.) shows a linear opaque radio-density around extraction socket in contrast with the experimental group (g.) which shows mottled trabecular pattern around extraction socket. Histological examination of ROI at 6 weeks after tooth extraction. Black arrows point to areas of inflammation (i.), yellow arrows to osteonecrosis area (k.). The experimental group exhibit areas with fewer bone lacunae and areas of dead bone, the control group shows bone deposition and presence of osteocytes in an organized bone matrix (h.j.).

In general, study animals tolerated the procedure well. Furthermore, animals showed suitable hemostasis and rapid recovery and no associated lesions were observed post anesthesia. (Fig. 3a,b)

Surgical procedure in the experimental group was longer than in the control group. Furthermore, dental fractures occurred during the extractions, resulting in the presence of residual root tips and fracture of the alveolar lamina or teeth extracted along with interradicular bone ( Table 1).

**Table 1:**
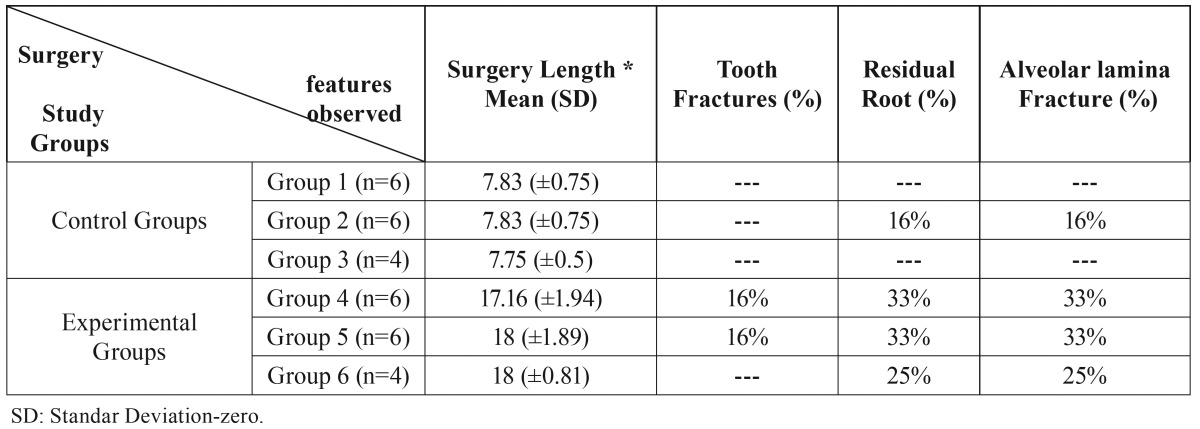
Surgery features * time, minutes.

- Macroscopic Analysis

In the experimental groups, rats showed ONJ, while in the control groups, the dental socket, was epithelialized although some areas exhibited accumulation of detritus. In the experimental groups, we observed dental opened socket; large areas of exposed bone as well as fractures. Furthermore, at the sixth week, we observed open sockets with large areas of exposed bone in the three experimental animals, while the control groups showed a healing dental socket cover it by epithelium. (Fig 3c,d).

Analysis of the alveolar characteristics was performed using Xi2 tests and showed that the differences between the experimental and control groups were statistically significant (*p*<0.05).

- Radiographic Analysis

This analysis revealed the presence of osteolytic lesions accompanied by extensive destruction and formation of sequestrum in experimental groups. In the control group, the sockets were nearly filled with mineralized connective tissue exhibiting normal trabecular patterns. In contrast, radiographs from animals treated with ZA displayed a lack of bone formation in alveoli (Fig 3f-g). Statistically, signiﬁcant differences were found in bone density between groups (*p*<0.05) ( Table 2).

**Table 2:**
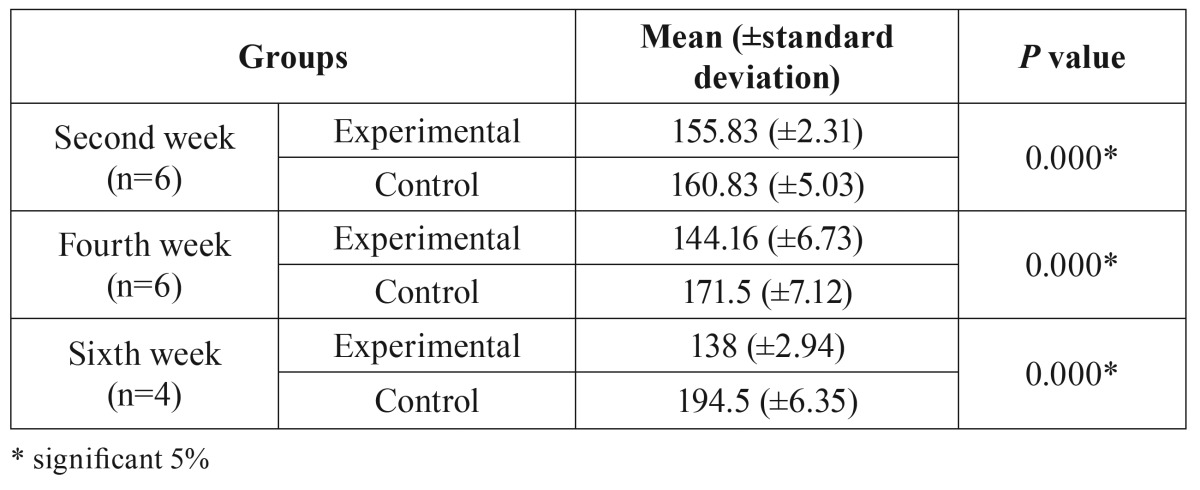
Gray level (GL) values in ROI analyzed with Student’s t-test between groups with 5% significance level (*P*<0.05).

- Histological Analysis

Histologically, the control groups showed the normal course of healing in extraction sockets with increase mineralized connective tissue. Numerous differentiated osteoblasts and developing a bone matrix with a high percentage of osteocytes appeared on the lining of the socket walls and expanding to the central region of the socket with a centripetal pattern, and complete mucosal coverage. Experimental groups showed extensive ulcerative lesion accompanied by exposed and necrotic bone with sequestrum and bacterial colonies. Inflammatory cell infiltration in the connective tissue was equally observed at first weeks. The morphometric analysis showed that the experimental groups had lower osteoclast number than each control group (*p*<0.05), also showed higher necrotic bone fractions and demonstrated significantly higher empty lacunae (*p*<0.05). The control groups showed bone matrix with highly eosinophilic and organized (Fig. 3). In contrast, the experimental group exhibited scant, poorly organized bone matrix ( Table 3).

**Table 3:**
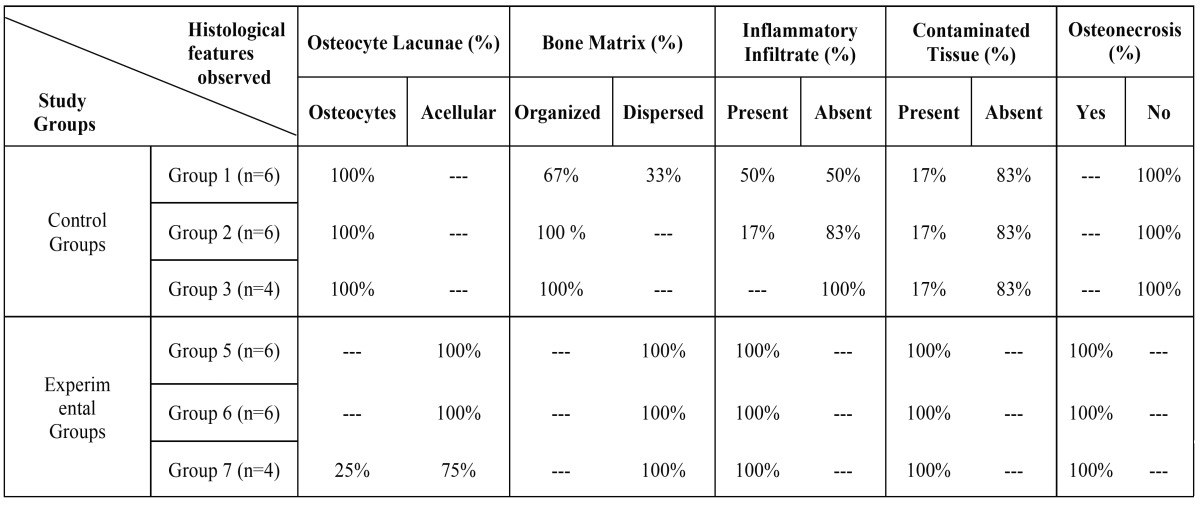
Histological Examination.

## Discussion

Currently, it is necessary to obtain an animal model for a better understanding of pathophysiology and to develop standards for the prevention and treatment of BRONJ.

The aim of our study was to develop a clinically relevant, inexpensive, and easily replicable animal model affected by BRONJ.

We found that a short lapse dose of BP before the surgical procedure develops BRONJ while other studies employed high doses and over longer periods of time (14,18,19).

To assess the speciﬁc effects of bisphosphonate on the alveolar bone tissue, we treated our animals in the absence of other diseases or medications. In contrast, other studies concerning BRONJ have changed the metabolic conditions of rats by using additional bisphosphonate’s therapies with immunosuppressant drugs ( Table 4).

**Table 4:**
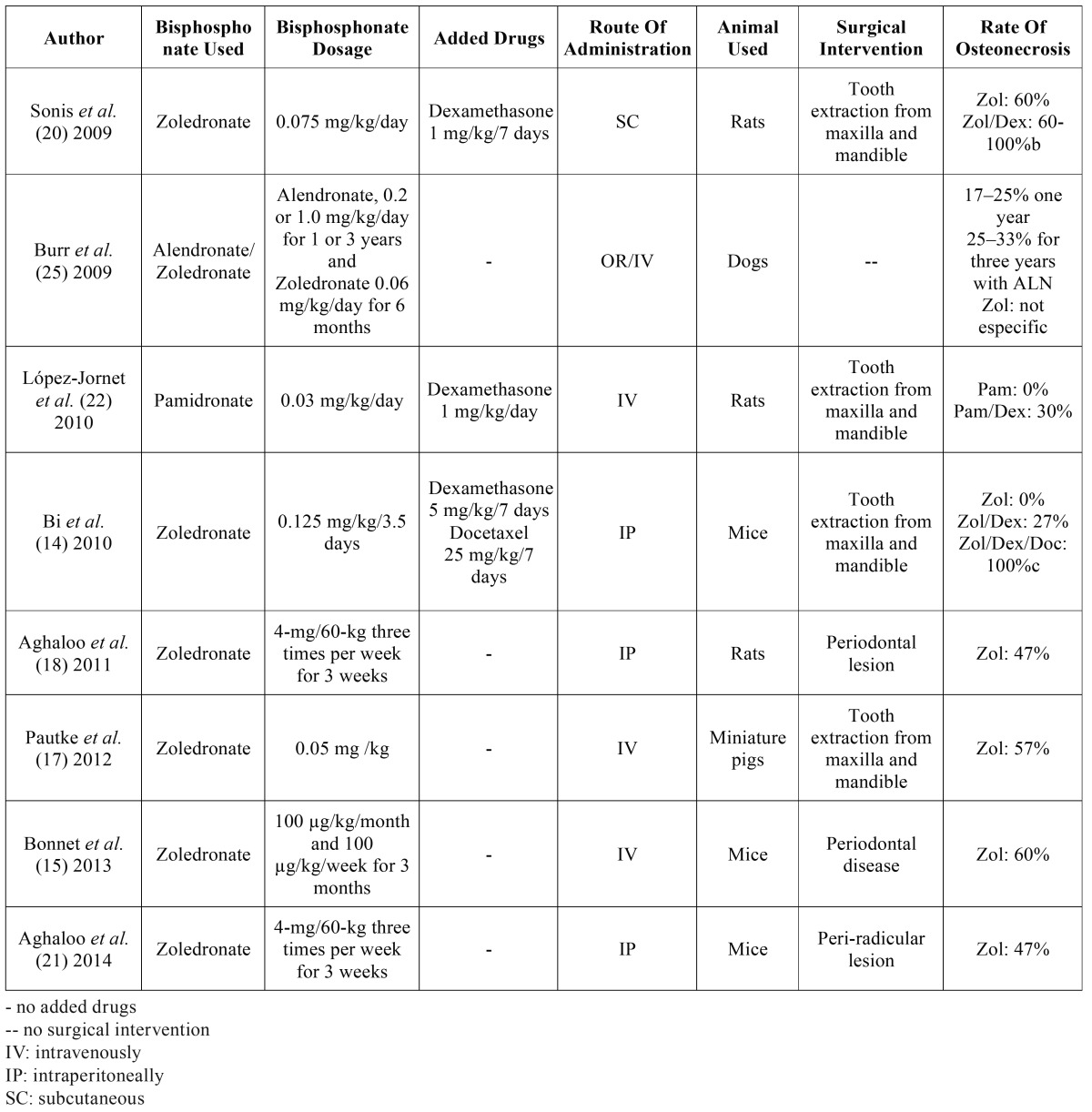
Animal models to study osteonecrosis of the jaw related to bisphosphonate treatment.

The dental extraction is the strongest risk factor for the development of BRONJ in patients receiving bisphosphonates. By extracting only the second maxillary molar we were able to produce BRONJ in the experimental group (See Fig. 3) (17,15,20-22), while previous studies had extracted all hemimandible molars to obtain the same result (20,22).

The rats receiving bisphosphonates showed more surgical complications than the control group ( Table 1). Our results demonstrated that the usage of zoledronic acid increases the alveolar bone density. This condition makes the surgical procedures difficult and they require a longer time than usual. We also observed that the experimental group exhibit evident delayed reepithelialization at the fourth week, in contrast with other BRONJ models which showed the same condition but during a longer period of time. (2,9,22,23)

The X-Ray results showed no overlapping structures, radiolucent areas with a lack of bone formation (EG). These data were analyzed considering the grey-level proﬁles to demonstrate quantitatively assessed bone density in the alveolar area. Several other studies have used X-Ray and micro CT, but this work did quantitative analyses and not only descriptive ones (11,14,17).

We agree in our histological analysis with other studies. The experimental groups show decreased bone lacunae, osteoclastic bone surfaces, a reduced bone formation rate and severely affected bone matrix (9,23,24).

The previous animal studies have reported different rates for BRONJ development ( Table 4). The success rate observed in the present study must be attributed to several factors, including the use of a high dose of zoledronic acid administered in a short time lapse, and also, the sacrifice time is not extended to show BRONJ.

This study has several strengths, including BRONJ development in a short period, because we have significant results at four weeks after dental extraction, thus reducing the suffering of animals. The high availability, inexpensiveness, and easy handling of this animal model make it an excellent option for studies to research BRONJ pathophysiology.

Our rat model will allow contributing to conduct prospective controlled studies to evaluate the different risk factors, etiology, prevention and treatment of BRONJ. Additionally, this animal model provides an additional useful, practical, and cost-effective model to develop new therapeutic strategies to cure this particular side effects in human.

Recently, a new side effect has been described, Medication-Related Osteonecrosis in Jaws (MROJ). We appreciate that our study is limited to one type of antiresorptive medication, acid zoledronic. However, it is enough to develop BRONJ. Thus, our research group considers that is necessary to develop animal models using other antiangiogenic and antiresorptive agents such as denosumab and bevacizumab.

Finally, in the model involving the extraction of the second maxillary molar of rats treated with two zoledronic acid doses of 0.06mg/kg per two weeks, osteonecrosis lesions were found in all cases. The model has to be used to analyze the changes occurring in the tissues involved in the extraction at around six weeks; it is no necessary the extension of the study beyond this time. BRONJ studies have been based on case reviews and animal studies.

## References

[1] Campisi G, Fedele S, Fusco V, Pizzo G, Di Fede O, Bedogni A (2014). Epidemiology, clinical manifestations, risk reduction and treatment strategies of jaw osteonecrosis in cancer patients exposed to antiresorptive agents. Future Oncol.

[2] Landesberg R, Woo V, Cremers S, Cozin M, Marolt D, Vunjak-Novakovic G (2011). Potential pathophysiological mechanism in osteonecrosis of the jaw. Ann N Y Acad Sci.

[3] Berti-Couto SA, Vasconcelos AC, Iglesias JE, Figueiredo MA, Salum FG, Cherubini K (2014). Diabetes mellitus and corticotherapy as risk factors for alendronate-related osteonecrosis of the jaws: a study in wistar rats. Head Neck.

[4] Coskun Benlidayi I, Guzel R (2013). Oral bisphosphonate related osteonecrosis of the jaw: a challenging adverse effect. ISRN Rheumatol.

[5] Ruggiero SL, Mehrotra B, Rosenberg TJ, Engroff SL (2004). Osteonecrosis of the jaws associated with the use of bisphosphonates: a review of 63 cases. J Oral Maxillofac Surg.

[6] Colella G, Campisi G, Fusco V (2009). American Association of Oral and Maxillofacial Surgeons position paper: Bisphosphonate-related osteonecrosis of the jaws-2009 update: the need to refine the BRONJ definition. J Oral Maxillofac Surg.

[7] Ruggiero SL, Dodson TB, Fantasia J, Goodday R, Aghaloo T, Mehrotra B (2014). American Association of Oral and Maxillofacial Surgeons. American Association of oral and Maxillofacial Surgeons position paper on medication-related osteonecrosis of the jaw-2014. J Oral Maxillofac Surg.

[8] Zhang X, Hamadeh IS, Song S, Katz J, Moreb JS, Langaee TY (2016). Osteonecrosis of the jaw in the United States Food and Drug Administration's adverse event reporting system (FAERS). J Bone Miner Res.

[9] Pacheco VN, Langie R, Etges A, Ponzoni D, Puricelli E (2015). Nitrogen-containing bisphosphonate therapy: assessment of the alveolar bone structure in rats - a blind randomized controlled trial. Int J Exp Pathol.

[10] Hamadeh IS, Ngwa BA, Gong Y (2015). Drug induced osteonecrosis of the jaw. Cancer Treat Rev.

[11] Kozloff KM, Volakis LI, Marini JC, Caird MS (2010). Near-infrared fluorescent probe traces bisphosphonate delivery and retention in vivo. J Bone Miner Res.

[12] Ali-Erdem M, Burak-Cankaya A, Cemil-Isler S, Demircan S, Soluk M, Kasapoglu C (2011). Extraction socket healing in rats treated with bisphosphonate: animal model for bisphosphonate related osteonecrosis of jaws in multiple myeloma patients. Med Oral Patol Oral Cir Bucal.

[13] Biasotto M, Chiandussi S, Zacchigna S, Moimas S, Dore F, Pozzato G (2010). A novel animal model to study non- spontaneous bisphosphonate necrosis of jaw. J Oral Pathol Med.

[14] Bi Y, Gao Y, Ehirchiou D, Cao C, Kikuiri T, Le A (2010). Biphosphonate cause osteonecrosis of the jaw-like disease in mice. Am J Pathol.

[15] Bonnet N, Lesclous P, Saffar JL, Ferrari S (2013). Zoledronate effects on systemic and jaw osteopenias in ovariectomized periostin-deficient mice. PLoS One.

[16] de Molon RS, Cheong S, Bezouglaia O, Dry SM, Pirih F, Cirelli JA (2014). Spontaneous osteonecrosis of the jaws in the maxilla of mice on antiresorptive treatment: a novel ONJ mouse model. Bone.

[17] Pautke C, Kreutzer K, Weitz J, Knödler M, Münzel D, Wexel G (2012). Biphosphonate related osteonecrosis of the jaw: A minipig large animal model. Bone.

[18] Aghaloo TL, Kang B, Sung EC, Shoff M, Ronconi M, Gotcher JE (2011). Periodontal disease and bisphosphonates induce osteonecrosis of the jaws in the rat. J Bone Miner Res.

[19] Aguirre JI, Altman MK, Vanegas SM, Franz SE, Bassit ACF, Wronski TJ (2010). Effects of alendronate on bone healing after tooth extraction in rats. Oral Dis.

[20] Sonis ST, Watkins BA, Lyng GD, Lerman MA, Anderson KC (2009). Bony changes in the jaws of rats treated with zoledronic acid and dexamethasone before dental extractions mimic bisphosphonate-related osteonecrosis in cancer patients. Oral Oncol.

[21] Aghaloo TL, Cheong S, Bezouglaia O, Kostenuik P, Atti E, Dry SM (2014). RANKL inhibitors induce osteonecrosis of the jaw in mice with periapical disease. J Bone Miner Res.

[22] López-Jornet P, Camacho-Alonso F, Molina-Miñano F, Gómez-García F, Vicente-Ortega V (2010). An experimental study of bisphosphonate-induced jaws osteonecrosis in Sprague–Dawley rats. J Oral Pathol Med.

[23] Jang HW, Kim JW, Cha IH (2015). Development of animal model for Bisphosphonates-related osteonecrosis of the jaw (BRONJ). Maxillofac Plast Reconstr Surg.

[24] Odvina CV, Zerwekh JE, Rao DS, Maalouf N, Gottschalk FA, Pak CYC (2005). Severely Suppressed Bone Turnover: A Potential Complication of Alendronate Therapy. J Clin Endocrinol Metab.

[25] Burr DB, Allen MR (2009). Mandibular necrosis in beagle dogs treated with bisphosphonates. Orthod Craniofac Res.

